# The intestinal stem cell/enteroblast-GAL4 driver, *escargot-GAL4*, also manipulates gene expression in the juvenile hormone-synthesizing organ of *Drosophila melanogaster*

**DOI:** 10.1038/s41598-024-60269-2

**Published:** 2024-04-26

**Authors:** Yoshitomo Kurogi, Yosuke Mizuno, Takumi Kamiyama, Ryusuke Niwa

**Affiliations:** 1 Graduate School of Science and Technology, University of Tsukuba, Ibaraki, 305-8577 Japan; 2 Life Science Center for Survival Dynamics, Tsukuba Advanced Research Alliance (TARA), University of Tsukuba, Ibaraki, 305-8577 Japan

**Keywords:** Genetics, Gene expression, Animal physiology, Genetic techniques

## Abstract

Intestinal stem cells (ISCs) of the fruit fly, *Drosophila melanogaster*, offer an excellent genetic model to explore homeostatic roles of ISCs in animal physiology. Among available genetic tools, the *escargot* (*esg*)*-GAL4* driver, expressing the yeast transcription factor gene, *GAL4*, under control of the *esg* gene promoter, has contributed significantly to ISC studies. This driver facilitates activation of genes of interest in proximity to a GAL4-binding element, Upstream Activating Sequence, in ISCs and progenitor enteroblasts (EBs). While *esg-GAL4* has been considered an ISC/EB-specific driver, recent studies have shown that *esg-GAL4* is also active in other tissues, such as neurons and ovaries. Therefore, the ISC/EB specificity of *esg-GAL4* is questionable. In this study, we reveal *esg-GAL4* expression in the *corpus allatum* (CA), responsible for juvenile hormone (JH) production. When driving the oncogenic gene, *Ras*^*V12*^, *esg-GAL4* induces overgrowth in ISCs/EBs as reported, but also increases CA cell number and size. Consistent with this observation, animals alter expression of JH-response genes. Our data show that *esg-GAL4*-driven gene manipulation can systemically influence JH-mediated animal physiology, arguing for cautious use of *esg-GAL4* as a “specific” ISC/EB driver to examine ISC/EB-mediated animal physiology.

## Introduction

Precise overexpression of genes in specific cell types and time windows is crucial to discover essential functions of those genes in multicellular organisms. Among model organisms, such gene expression manipulation techniques are best developed for the fruit fly, *Drosophila melanogaster*. In particular, the GAL4-UAS system is a powerful binary gene expression system in *D. melanogaster* for targeted genetic manipulation in a spatio-temporal specific manner to reveal gene functions^1^. This system utilizes the yeast transcription factor, GAL4, controlled by a tissue-specific enhancer/promoter sequence, in combination with a GAL4-biding element called Upstream Activating Sequence (UAS), inserted upstream of the gene of interest, either endogenously or exogenously. The impact of the GAL4-UAS system on *D. melanogaster* genetics research is immeasurable. However, despite its utility, a potential drawback of the GAL4-UAS system is the possibility of incomplete cell type- or tissue-specific expression patterns, complicating interpretation of results.

**Figure 1:**
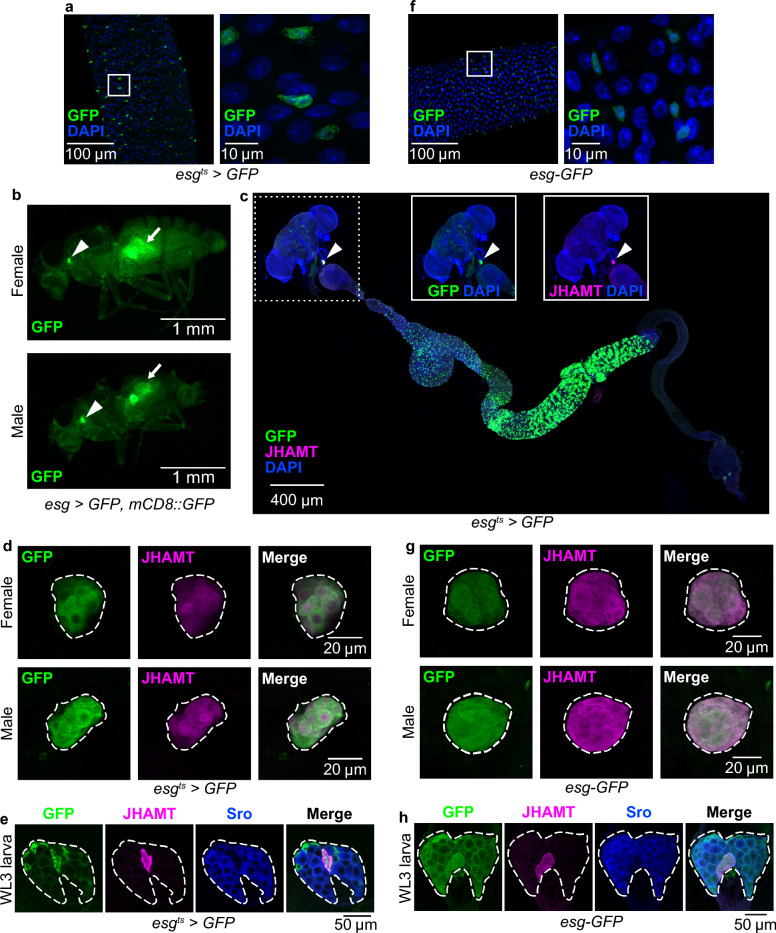
The transcription factor encoding gene, *esg*, was expressed in the CA. (**a**) (Left) *GFP* (green) driven by *esg-GAL4* with *tubP-GAL80*^*ts*^ (*esg*^*ts*^ >) was expressed in a subpopulation of adult midgut cells. (Right) magnified view of the area enclosed by the white square in the left figure. Blue is the DAPI signal. (**b**) *esg* > *GFP, mCD8::GFP* expression in whole bodies of adult males and females. Arrowheads and arrows indicate the CA and midguts, respectively. (**c**) *esg*^*ts*^ > *GFP* (green) was expressed not only in some midgut cells, but also in the CA (arrowhead). This sample was derived from a female. The CA was labeled with anti-JHAMT antibody (magenta). Two inset images correspond to a region marked with a dashed line surrounding the brain, CA, and proventriculus. Both *esg*^*ts*^ > *GFP* and anti-JHAMT immunoreactive signals were observed in the CA. (**d**) *GFP* (green) driven by *esg-GAL4* labeled the CA in both adult males (upper) and females (lower). The CA was labeled with anti-JHAMT antibody (magenta). (**e**) *GFP* (green) driven by *esg-GAL4* labeled the CA and a part of the prothoracic gland (PG) in wandering 3rd-instar (WL3) larva. The CA was labeled with anti-JHAMT antibody (magenta) and the PG was labeled with anti-Shroud (Sro) antibody (blue). (**f**) (Left) *esg-knock in-GFP* (green) was expressed in a subpopulation of adult midgut cells. (Right) A magnified view of the area encircled with a white line in the left figure. Blue is the DAPI signal. (**g**) *esg-knock in-GFP* (*esg-GFP*, green) was expressed in the CA in both males (upper) and females (lower). The CA was labeled with anti-JHAMT antibody (magenta). (**h**) *esg-knock in-GFP* (green) was expressed in the CA in WL3 larvae. The CA was labeled with anti-JHAMT antibody (magenta) and the PG was labeled with anti-Sro antibody (blue).

*D. melanogaster escargot (esg)-GAL4*, formally known as *P{GawB}NP5130* (RRID:BDSC_93857)^2^, has widely been used as the fundamental GAL4 driver to manipulate genes “specifically” in intestinal stem cells (ISCs) and enteroblasts (EBs) (Fig. 1a). In *D. melanogaster*, ISCs regulate gut homeostasis by maintaining themselves and also by giving rise to other essential gut epithelial cells, including EBs, enteroendocrine cells, and enterocytes. Dysfunction of ISCs results in severe malfunctions of age-associated tissue integrity in the gut^3^. By virtue of convenient tools to analyze functions and roles of genes, *D. melanogaster* ISCs have served as a useful model system to study the homeostatic role of ISCs in gut physiology. Notably, the *esg-GAL4* driver has facilitated overexpression of genes and various constructs to study fundamental roles of ISCs and EBs. For example, researchers have heavily used *esg-GAL4* to generate ISC tumors by overexpressing oncogenic genes such as the gain-of-function transgenes, *Ras* and *yokie (yki)*. *esg-GAL4*-driven ISC tumor animals have advanced our understanding of tumor-dependent impairment of systemic physiology, such as cachexia and the bloating phenotype. The crucial assumption for interpreting results of these studies as a phenotype originating from ISCs and EBs is that *esg-GAL4* manipulates gene expression only in these cells in adults. However, recent studies reported that *esg-GAL4* is expressed at least in some brain neurons^4^, ovaries^5,6^, and Malpighian tubules^7^, but not in muscles or fat bodies^4^. Therefore, the ISC/EB-specificity of *esg-GAL4* has been questioned.

In this study, we report that *esg-GAL4* is also expressed in the insect endocrine organ, the *corpus allatum* (CA), which is essential for synthesizing insect juvenile hormone (JH). Our data show that *esg-GAL4*-driven gene manipulation can systemically influence JH-mediated animal physiology, arguing for cautious use of *esg-GAL4* as a “specific” ISC/EB driver to examine ISC/EB-mediated animal physiology.

## Result

### *Esg-GAL4* is expressed in the endocrine *corpus allatum*

We conducted experiments using the *esg-GAL4* driver combined with *tubulin* promoter-driven temperature-sensitive *GAL80* (*tubP-GAL80*^*ts*^). Hereafter, *esg-GAL4; tubP-GAL80*^*ts*^ is designated “*esg*^*ts*^*-GAL4”* or “*esg*^*ts*^ > ”. This strain has widely been used for adult stage-specific gene manipulation in ISCs and EBs^5,8–10^. In all experimental conditions in this study, we reared *esg*^*ts*^ > flies at a permissive temperature (21 °C) during development, such that *esg-GAL4* activity is suppressed by GAL80 right before eclosion. Then, after eclosion, we subjected these flies to a restrictive temperature (29 °C) to activate *esg-GAL4* only in the adult stage.

We realized by chance that *esg-GAL4* was active in the tissue located at the most anterior part of the thorax in both males and females (Fig. 1b). More precisely, *esg*^*ts*^*-GAL4*-positive tissue was observed between the brain and proventriculus (Fig. 1c). This tissue was co-immunostained with an antibody against juvenile hormone acid *O*-methyltransferase (JHAMT), the essential enzyme that synthesizes JH in the CA^11,12^. This result strongly indicates that *esg*^*ts*^*-GAL4*-positive tissue between the brain and proventriculus is the CA. We also confirmed that *esg*^*ts*^*-GAL4* was expressed in the CA of both male and female adult flies (Fig. 1d). Moreover, *esg*^*ts*^*-GAL4* was expressed in the ring gland, particularly in the CA of wandering 3rd-instar larvae, as well as of adults (Fig. 1e). These results suggest that *esg*^*ts*^*-GAL4* labels the CA in both male and female larvae and adults.

To confirm whether the *esg* gene itself is expressed in the CA, we used the *esg-knock-in-GFP* (*esg-GFP*) line^13^. As with *esg*^*ts*^ > *GFP* expression, *esg-GFP* was expressed not only in a certain cells in the midgut, which seem to be ISCs/EBs (Fig. 1f)^13^, but also in the CA of both adult males and females (Fig. 1g). Furthermore, *esg-GFP* was expressed in the CA of wandering 3rd-instar larvae, while considerable expression of *esg-GFP* was also detected in other ring gland cells (Fig. 1h). These results suggest that *esg* is endogenously expressed in the CA.

### RNAi of JH-biosynthetic enzyme by *esg-GAL4* also impairs oogenesis

**Figure 2:**
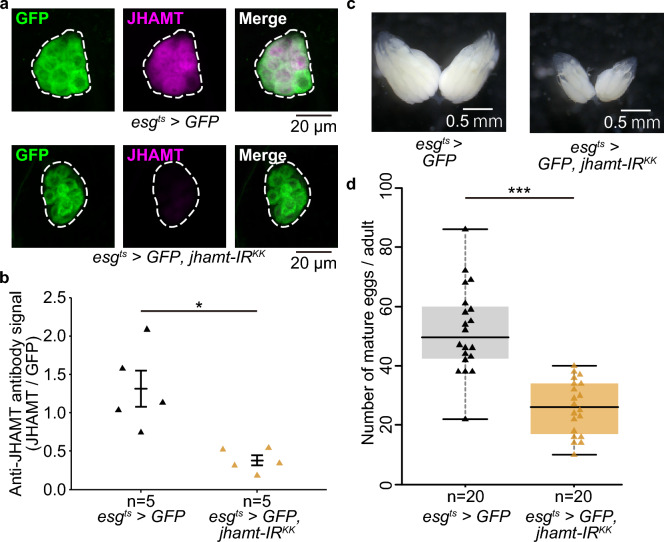
*jhamt*-RNAi driven by *esg-GAL4* inhibited *jhamt* in the CA and reduced the number of eggs in the ovary. (**a**) Immunostaining signal of anti-JHAMT (magenta) in the CA was drastically decreased by *jhamt*-RNAi, driven by *esg-GAL4*. Upper panels are controls (*esg*^*ts*^ > *GFP*) and lower panels are *jhamt*-RNAi (*esg*^*ts*^ > *GFP, jhamt-IR*^*KK*^) flies. The CA is encircled with a dashed line. (**b**) Quantification of anti-JHAMT antibody immunostaining signals normalized by the GFP signal between control and *jhamt*-RNAi flies. (**c**) Ovaries of control (upper: *esg*^*ts*^ > *GFP*) and *jhamt*-RNAi (lower: *esg*^*ts*^ > *GFP, jhamt-IR*^*KK*^) virgin females. (**d**) Numbers of mature eggs in virgin females were significantly decreased by adult-specific *jhamt-*RNAi driven by *esg-GAL4*. For each experimental condition, 20 adult females were used. The Wilcoxon rank sum test was used for these data. *P < 0.05, **P < 0.01, ***P < 0.001.

Next, we explored the possibility that *esg*^*ts*^*-GAL4-*driven transgenic RNAi suppresses gene expression in the CA. To examine this point, we conducted an RNAi experiment with *esg*^*ts*^*-GAL4* to target *jhamt*, which is expressed explicitly in the CA^12^. Immunostaining signals of anti-JHAMT antibody were drastically decreased by *jhamt* RNAi, compared to controls (Fig. 2a,b; Supplementary Table S1).

In many insects, including *D. melanogaster*, JH promotes ovarian development by accumulating yolk components such as yolk protein and vitellogenin^14,15^. In *D. melanogaster*, a previous study reported that loss of *jhamt* activity results in smaller ovaries and reduced egg numbers^16^. Therefore, we observed ovary morphology and counted numbers of mature eggs in adult females expressing *esg*^*ts*^ > *jhamt* RNAi. We found that *esg*^*ts*^ > *jhamt* RNAi flies had smaller ovaries than controls (Fig. 2c). Consistent with this observation, the number of mature eggs was significantly decreased in RNAi flies (Fig. 2d; Supplementary Table S1). These results suggest that *esg*^*ts*^*-GAL4*-driven RNAi suppresses gene expression in the CA and influences JH-mediated biological events such as oogenesis.

### Oncogenic *Ras*^*V12*^ expression by *esg-GAL4* causes CA hypertrophy and abnormal expression of JH-responsive genes

**Figure 3:**
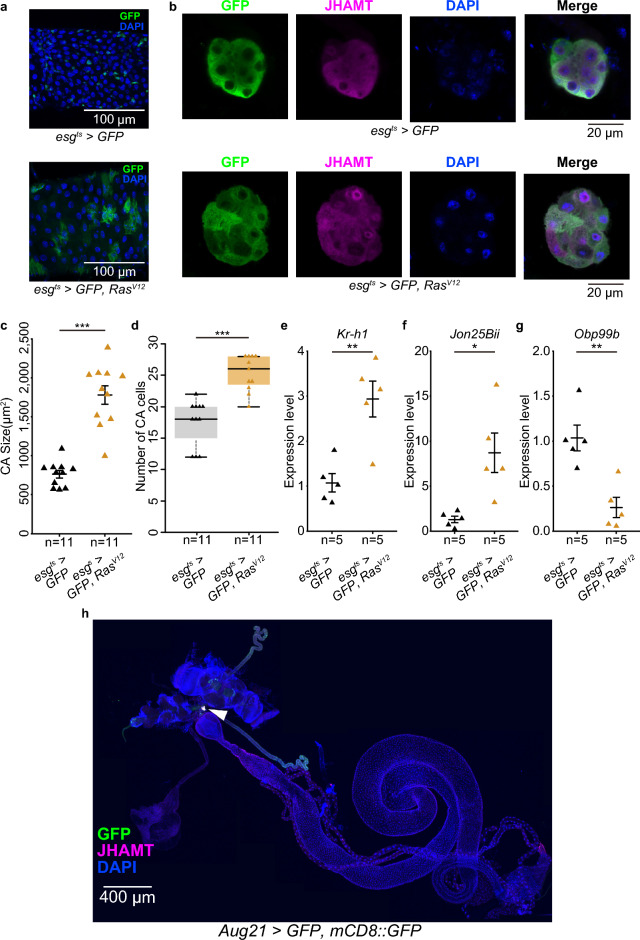
*Ras*^*V12*^ driven by *esg-GAL4* caused hypertrophy of the CA. (**a**) Ectopic expression of *RasV*^*12*^ induced overproliferation of *esg-GAL4* positive midgut cells (GFP: green). (**b**) The CA of control (*esg*^*ts*^ > *GFP*) adult virgin females (upper) and *RasV*^*12*^ overexpression (*esg*^*ts*^ > *GFP, RasV*^*12*^) adult virgin females (lower). The same laser power and software gain were used to image intestinal or CA fluorescence, regardless of genotype. (**c**) The CA was enlarged by adult-specific *RasV*^*12*^ overexpression. (**d**) Numbers of CA cells were increased by adult-specific *RasV*^*12*^ overexpression. (**e**–**g**) Expression levels of JH-responsive genes (**e**: *Kr-h1*, **f**: *Jon25Bii*, **g**: *Obp99b*) were significantly changed by adult-specific *RasV*^*12*^ overexpression. N means sample size. The Wilcoxon rank sum test was used for (**c**,**d**). Student’s* t*-test was used for (**e**–**g**). *P < 0.05, **P < 0.01, ***P < 0.001. (**h**) *Aug21* > *GFP, mCD8::GFP* (green) was expressed in the CA (arrowhead), but not in midgut cells. This sample was derived from a female. The CA was labeled with anti-JHAMT antibody (magenta). Blue is the DAPI signal.

In some recent studies, *esg*^*ts*^*-GAL4* and *UAS-Ras*^*V12*^ have been utilized to induce ISC/EB tumors to investigate cell turnover in the midgut and tumor-mediated systemic physiology^8,17,18^. Since *esg-GAL4* is also expressed in the CA, *esg*^*ts*^ > *Ras*^*V12*^ might affect both ISC/EB and CA cells. Notably, *esg*^*ts*^ > *Ras*^*V12*^ resulted not only in abnormal expansion of the *esg*^*ts*^*-GAL4*-driven *GFP*-positive area in the midgut (Fig. 3a)^17^, but also increased CA size and cell number (Fig. 3b–d; Supplementary Table S2).

Considering morphological abnormalities in the CA, it seemed possible that *esg*^*ts*^ > *Ras*^*V12*^ expression enhances JH biosynthesis in the CA. Therefore, we next performed quantitative PCR on three JH-responsive genes, *Krüppel-homolog 1* (*Kr-h1*), *Jonah 25Bii* (*Jon25Bii*), *Odorant-binding protein 99b* (*Obp99b*), to estimate the amount of JH in *Ras*^*V12*^ overexpressors and controls^19^. Previous studies have shown that expression levels of *Kr-h1* and *Jon25Bii* correlate positively with the amount of JH in the body, while *Obp99b* correlates negatively^19,20^. Our qPCR results showed that expression levels of *Kr-h1* and *Jon25Bii* were increased by *Ras*^*V12*^, while *Obp99b* was decreased (Fig. 3e–g; Supplementary Table S2). These results strongly suggest that *esg*^*ts*^ > *Ras*^*V12*^ leads to abnormalities in CA cells and increased JH biosynthesis.

### The CA-driver *Aug21-GAL4* is not active in ISCs/EBs

A previous study has suggested that JH secreted from the CA is received by ISCs and/or EBs, enhancing ISC proliferation and leading to post-mating gut remodeling^5^. To overexpress genes to inhibit JH biosynthesis, the previous study utilized *Aug21-GAL4*, which is one of the most widely used CA-*GAL4* drivers^21^. Since *esg-GAL4* has high activity not only in ISCs/EBs, but also in the CA, we also verified whether *Aug21-GAL4* is active in ISCs/EBs, as well as in the CA. We found that *Aug21-GAL4*-driven GFP signals, as well as anti-JHAMT immunoreactive signals, were present in the CA, but not in ISCs, EBs, or other types of midgut cells (Fig. 3h). This observation suggests that *Aug21-GAL4*-mediated manipulation of gene expression does not directly affect gene expression in ISCs and EBs.

### Expression of other ISC/EB drivers in the CA

**Figure 4:**
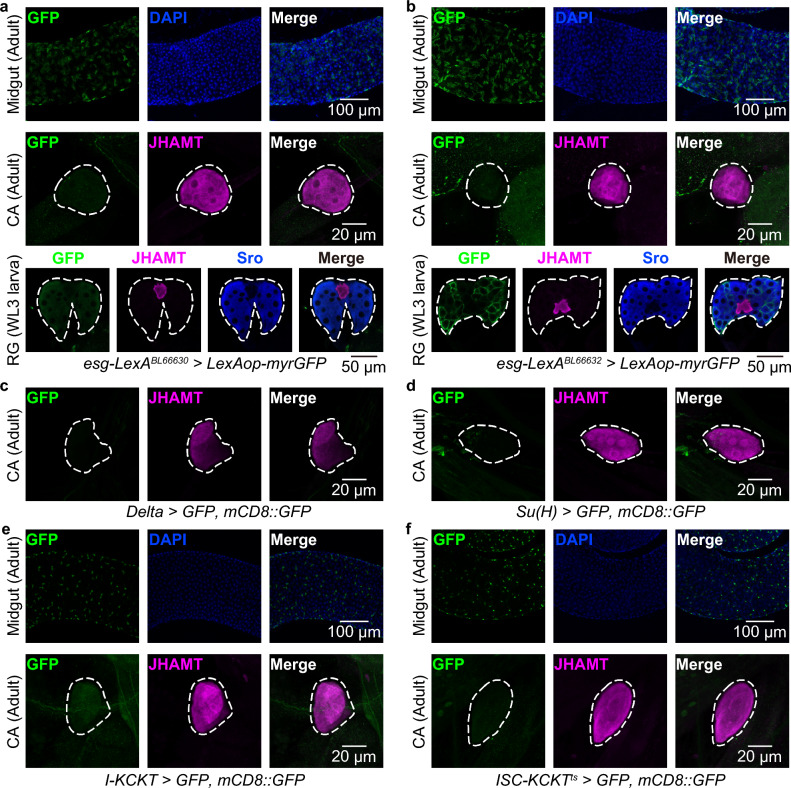
Expression of other ISC/EB drivers in the CA. *GFP* (green) driven by several drivers is shown. The CA was labeled with anti-JHAMT antibody (magenta). (**a**,**b**) *GFP* expression driven by two independent *esg-LexA* drivers in adult midguts (top), adult CA (middle), and ring glands (RG), containing the CA and PG, from wandering 3rd-instar (WL3) larvae (bottom). The PG was labeled with anti-Shroud (Sro) antibody (blue). (**c**,**d**) *GFP* expression driven by *Delta-GAL4* (**c**) and *Su(H)-GAL4* (**d**) in adult CA. (**e**,**f**) *GFP* expression driven by *I-KCKT-GAL4* (**e**) and *ISC-KCKT*^*ts*^*-GAL4* (**f**) in adult midguts (top) and CA (bottom).

Lastly, we examined whether other *GAL4* and *LexA* drivers used for gene manipulation in ISCs/EBs are also active in the CA. Beside *esg-GAL4*, many previous studies have used *esg-LexA* as an alternate binary gene expression driver active in ISCs/EBs^22–25^. We tested two *esg-LexA* drivers available from a stock center and found that both *esg-LexA* drivers were active in ISCs/EBs, but not in the adult or larval CA (Fig. 4a,b). It has also been reported that *Delta-GAL4* and *Su(H)-GAL4* are active in ISCs and EBs, respectively^26^. We found that the GFP signals driven by these GAL4 drivers were not present in the CA (Fig. 4c,d). These results suggest that the phenotypes resulting from genetic manipulation using these drivers are unlikely to be due to JH actions.

Another recent study reported new genetic tools, *intestinal-kickout (I-KCKT)-GAL4* and *ISC-KCKT*^*ts*^*-GAL4*, which are based in an intersectional method that restricts *GAL4* expression to ISCs and/or EBs^27^ (Fig. 4e,f). The study has confirmed that *I-KCKT-GAL4* and *ISC-KCKT*^*ts*^*-GAL4* are active in ISCs/EBs more specifically than conventional ISC/EB GAL4 drivers, such as *esg-GAL4*, *Delta-GAL4*, and *Su(H)-GAL4*, all of which are active in many types of cells other than ISCs/EBs^4,27^. We found that neither *I-KCKT-GAL4* nor *ISC-KCKT*^*ts*^*-GAL4* labeled the CA, suggesting that these *KCKT*-based *GAL4* lines do not affect CA gene expression.

## Discussion

In this study, we found that *esg-GAL4*, which is widely used to label midgut ISC/EB^2^, was also expressed in the CA. *esg*^*ts*^*-GAL4* manipulates and influences gene expression in the CA, as *esg*^*ts*^*-GAL4* > *jhamt* RNAi decreases mature egg formation, which is a typical phenotype of reduced JH titer. Whereas *esg-GAL4* is active in some ovarian cells^5,6^, it is reported by FlyBase (https://flybase.org/reports/FBgn0028841.html) that *jhamt* expression is not detected in the ovary. Therefore, the mature egg number phenotype by *esg*^*ts*^*-GAL4* > *jhamt* RNAi is due to the knock-down of *jhamt* expression in the CA but not the ovary. Moreover, *esg*^*ts*^ > *Ras*^*V12*^ caused CA hypertrophy and influenced JH-responsive gene expression (Fig. 3b–g), suggesting that *esg*^*ts*^ > *Ras*^*V12*^ increases JH biosynthesis.

Our data suggest that *esg* itself is expressed in the CA in both males and females of both larvae and adults. Enrichment of *esg* expression in the larval ring gland and the adult CA were suggested by a previous microarray analysis^28^ and a recent single-cell RNA-seq analysis^29^, respectively. *esg* encodes a Snail-type transcription factor that contributes to cell cycle regulation, cell differentiation, and cell–cell adhesion in many cell types in *D. melanogaster*^30–33^. However, functions of Esg that regulate differentiation and morphogenesis of the CA have not been studied. Thus, additional studies are needed to clarify how Esg is involved in CA cell regulation, especially whether it regulates JH biosynthesis.

In *D. melanogaster*, one of the reported functions of JH is that this hormone directly acts on ISCs and EBs through nuclear JH receptors, Methoprene-tolerant (Met) and Germ cell expressed (Gce), to regulate gut remodeling in mated or aged females^5,8^. Interestingly, previous studies reported that *esg*^*ts*^ > *jhamt* RNAi reduces numbers of ISCs and EBs. This phenotype is also observed in *esg*^*ts*^ > *Met* or *gce* RNAi animals. Since these studies use *esg*^*ts*^*-GAL4* as the ISC/EB- “specific” GAL4 driver, these papers propose that JH is biosynthesized in ISCs and EBs outside the CA, and cell-autonomously regulates maintenance of ISCs and EBs during aging^8^. However, our data strongly indicate that *esg-GAL4* is also expressed in the CA. More importantly, *esg*^*ts*^ > *jhamt* RNAi causes a decrease in JHAMT protein in the CA (Fig. 2a), which implies decreased JH biosynthesis in the CA, hence the systemic decrease in JH titer. We emphasize that although these previous studies did not examine *jhamt* expression in ISCs and EBs, they carefully evaluated functions of JHAMT in ISCs and EBs with additional experiments in which they utilize other GAL4 drivers to knock down *jhamt* in ISCs and EBs via *Delta-GAL4* and *Su(H)-GAL4*, respectively^8^. Therefore, we argue that JH biosynthesis most likely occurs in ISCs and EBs, although we cannot detect obvious anti-JHAMT reactivities in the gut region (Fig. 1c, 3h).

Nevertheless, a previous study using *Aug21-GAL4*, the well-known CA-*GAL4* driver^21^, revealed that *Aug21-GAL4*-mediated inhibition of JH biosynthesis in the CA suppresses ISC proliferation^5^. *Aug21-GAL4* is not active in ISCs/EBs (Fig. 3h), suggesting that the *Aug21-GAL4*-driven ISC phenotype cannot be explained by JH biosynthesis in ISCs/EBs. Taken together, we cannot rule out the possibility that JH is also supplied from the CA for maintenance of ISCs and EBs. In addition, ISC/EB increased by JH derived from the CA may further enhance JH production in the gut, implying that CA-ISC/EB interactions are more complex than previously thought. In the future, it will be important to investigate how much CA and ISC/EB each contribute to the circulating JH levels.

In the last decade, *esg-GAL4* has widely been used to generate ISC tumors by overexpressing oncogenic genes such as the gain-of-function transgenes, *Ras*, *Raf*, and *yki*^17,34^. In particular, very recently, a number of studies have utilized *esg-GAL4*-driven oncogenic gene models to study how ISC tumors impact gut homeostasis, as well as systemic physiology^35^. However, based on our results, when interpreting these *esg-GAL4*-driven phenotypes, we should consider not only effects of ISC/EB tumors, but also effects of JH biosynthesis abnormalities caused by CA hypertrophy. For example, some intestinal cells receive JH from the CA through Met and Gce, influencing gut remodeling^5,36^. Beside *Ras*^*V12*^, recent studies have shown that *esg*^*ts*^ > *yki*^*3SA*^ leads to severe cachexia and bloating phenotypes, mediated by abnormal hormone secretion from organs such as Malpighian tubules and midgut^9,23,37,38^. Considering the systemic nature of these ISC/EB tumor-associated phenotypes, it may be necessary to consider the function of JH, which has major impacts on insect physiology. Based on observations in this and a previous study^27^, *I-KCKT-GAL4* and *ISC-KCKT*^*ts*^*-GAL4* seem to be the current best choice of *GAL4* drivers to manipulate gene expression specifically in ISCs and EBs without influencing the CA. However, even *I-KCKT-GAL4* is active in Malpighian tubules^27^. Generally speaking, it will be important to examine phenotypes using more than just one *GAL4* driver.

## Methods

### *Drosophila* strains and maintenance

*D*. *melanogaster* was raised on a standard yeast-corn meal-glucose fly medium (0.275 g agar, 5.0 g glucose, 4.5 g cornmeal, 2.0 g yeast extract, 150 μL propionic acid, and 175 μL 10% butyl p-hydroxybenzoate (in 70% ethanol) in 50 mL water) at 25 ºC under a 12:12 h light/dark cycle.

Throughout this study, we used *esg*^*ts*^*-GAL4* flies (a gift from Fumiaki Obata, RIKEN Center for Biosystems Dynamics Research) that carried both *esg-GAL4* (RRID:BDSC_93857)^27^ and *tub-GAL80*^*ts*^. The following transgenic strains were also used: *Aug21-GAL4*^21^ (BDSC #30137), *Delta-GAL4*^26^ (a gift from Yuichiro Nakajima, University of Tokyo), *esg-GFP* (BDSC #78333), *esg-LexA*^*BL66630*^ ^22^ (BDSC #66630, a gift from Sa Kan Yoo, RIKEN Center for Biosystems Dynamics Research), *esg-LexA*^*BL66632*^^22^ (BDSC #66632, a gift from Sa Kan Yoo, RIKEN Center for Biosystems Dynamics Research), *I-KCKT-GAL4.p65*^27^ (BDSC #91526), *ISC-KCKT-GAL4*^*ts*^^27^ (BDSC #91411), *LexAop-myrGFP* (BDSC #32209), *Su(H)-GAL4*^26^ (a gifts from Yuichiro Nakajima, University of Tokyo), *UAS-GFP; UAS-mCD8::GFP*^39^ (a gift from Kei Ito, University of Cologne, Germany), *UAS*-*jhamt*-*IR*^KK^ (VDRC #103958), and *UAS-RasV12* (BDSC #4847). For adult-specific *GAL4* activation, flies carrying *esg*^*ts*^*-GAL4* were reared at 21 °C from embryos to newly eclosed adults. 0–12 h after eclosion, flies were moved to 29 °C. To visualize *esg*^*ts*^ > *GFP*, wandering 3rd-inster larvae were used. Larvae were reared at 25 °C until the middle 3rd-larval instar and transferred to 29 °C for 24 h before dissection. Heterozygous controls were obtained by crossing* w*^*1118*^ with strains of *GAL4* drivers.

### Immunohistochemistry

Tissues were dissected in PBS and fixed in 4% paraformaldehyde in PBS for 30–60 min at 25–27 °C. Fixed samples were rinsed thrice in PBS, washed for 15 min with PBS containing 0.3% Triton X-100 (Nacalai Tesque #35501-15) (PBT), and treated with a blocking solution (2% bovine serum albumin in PBT; Sigma #A9647) for 1 h at 25–27 °C or overnight at 4 °C. Samples were incubated with a primary antibody in blocking solution overnight at 4 °C. Primary antibodies used were as follows: chicken anti-GFP antibody (Abcam #ab13970, 1:2,000), guinea pig anti-JHAMT antibody (1:2,000)^40^, rabbit anti-JHAMT antibody (1:1,000)^12^, guinea pig anti-Shroud (Sro) antibody (1:400)^41^. Samples were rinsed thrice with PBS and then washed for 15 min with PBT, followed by incubation with fluorophore (Alexa Fluor 488, 555, and 633)-conjugated secondary antibodies (Thermo Fisher Scientific #A32931, #A21435, #A32732, and #A21105; 1:200), in blocking solution for 2 h at RT or overnight at 4 °C. Nuclear stains used in this study were 4ʹ,6-diamidino-2-phenylindole (DAPI; Sigma-Aldrich #D9542; final concentration 1 μg/ml). For DAPI staining, after incubation with secondary antibodies, samples were washed and then incubated with DAPI for 1 h. After another round of washing, all samples were mounted on glass slides using FluorSave reagent (Merck Millipore, #345789). All fluorescence images were captured using Nikon SMZ25 dissection microscope with the imaging software NIS-Elements version 5.42.01 (for Fig. 1b) and Carl Zeiss LSM700 or LSM900 confocal microscopy with the imaging software Zen version 8,1,0,484 (for all other figures). Quantification of immunostaining signals was conducted using ImageJ software version 1.53q^42^.

### Counting mature egg numbers in ovaries

Virgin females carrying *esg*^*ts*^*-GAL4* were reared at 21 °C from embryos to newly eclosed adults. 0–12 h after eclosion, flies were transferred to 29 °C and reared for 4 days. In each vial, fewer than 10 females were reared. Ovaries of virgin females were dissected in PBS. Numbers of mature eggs (stage-14 oocytes)^43^ in the ovaries were counted under a stereomicroscope (Leica MZ10F).

### Reverse transcription-quantitative PCR (RT-qPCR)

Total RNA was extracted from whole bodies of 4-day-old adult virgin female flies. RNA was reverse-transcribed using ReverTra Ace qPCR RT Master Mix with gDNA Remover (TOYOBO #FSQ-301). Synthesized cDNA samples were used as templates for quantitative PCR using THUNDERBIRD SYBR qPCR Mix (TOYOBO #QPS-201) on a Thermal Cycler Dice Real Time System (Takara Bio #TP870). The amount of target RNA was normalized to the endogenous control *ribosomal protein 49* gene (*rp49*) and the relative change was calculated. Expression levels of each gene were compared using the ΔΔCt method. The following primers were used for this analysis: *rp49* F (5ʹ-CGGATCGATATGCTAAGCTGT-3ʹ), *rp49* R (5ʹ-GCGCTTGTTCGATCCGTA-3ʹ), *kr-h1* F (5ʹ-TCACACATCAAGAAGCCAACT-3ʹ), *kr-h1* R (5ʹ-GCTGGTTGGCGGAATAGTAA-3ʹ), *obp99b* F (5ʹ-AGCACGGATTCGATGTCCACAAGA-3ʹ), *obp99b* R (5ʹ-TTGGAGTTCATGAAGCACATGCCG-3ʹ), *jon25Bii* F (5ʹ-CAGGCTCAGTACACCCACAC-3ʹ), *jon25Bii* R (5ʹ-TGGTGTTGTAGTCCGAGTGC-3ʹ).

### Statistical analysis

All experiments were performed independently at least twice. Sample sizes were chosen based on the number of independent experiments required for statistical significance and technical feasibility. Experiments were not randomized, and investigators were not blinded. All statistical analyses were performed using “R”, software version 4.0.3. Details of statistical analyses are described in figure legends.

### Supplementary Information

Supplementary Tables S1 and S2.

## Data Availability

All numerical data are available in Supplementary Tables S1 and S2. All other data are available upon request to R.N.
